# SANS (USH1G) Molecularly Links the Human Usher Syndrome Protein Network to the Intraflagellar Transport Module by Direct Binding to IFT-B Proteins

**DOI:** 10.3389/fcell.2019.00216

**Published:** 2019-10-04

**Authors:** Nasrin Sorusch, Adem Yildirim, Barbara Knapp, Julia Janson, Wiebke Fleck, Caroline Scharf, Uwe Wolfrum

**Affiliations:** Molecular Cell Biology, Institute of Molecular Physiology, Johannes Gutenberg University Mainz, Mainz, Germany

**Keywords:** Usher syndrome, USH1G, IFT, ciliary transport, photoreceptor cell, primary cilia, ciliopathy, USH interactome

## Abstract

The human Usher syndrome (USH) is a retinal ciliopathy, characterized by profound congenital deafness, variable vestibular dysfunction and pre-pubertal onset of retinitis pigmentosa. In the effected sensory cells, USH protein networks are assumed to function in ciliary transport processes. The USH1G protein SANS is a scaffold of the ciliary/periciliary USH protein network of photoreceptor cells. Moreover, SANS is associated with microtubules, the transport routes for protein delivery toward the cilium. To enlighten the role of SANS in ciliary transport processes, we aimed to identify transport related proteins associated with SANS. The intraflagellar transport (IFT) system is a conserved mechanism for bi-directional transport toward and through primary cilia. Thus, we tested the direct binding of SANS to IFT molecules, namely IFT20, IFT57, and IFT74 in 1:1 yeast-two-hybrid assay. The identified SANS-IFT interactions were validated *in vitro* via independent complementary interaction assays and in cells by applying membrane targeting assays. Quantitative immunofluorescence microscopy revealed the co-localization of SANS with IFT20, IFT52, and IFT57 particularly at ciliary base of wild type mouse photoreceptor cells. Analysis of photoreceptor cells of SANS knock out mice revealed the decrease of IFTs in the ciliary compartment indicating a role of SANS in the proper positioning of IFT-B molecules in primary cilia. Our study demonstrated direct binding of IFT complex B proteins IFT52 and IFT57 to the N-terminal ankyrin repeats and the central domain of SANS. Our data also indicate that pathologic mutations in the N-terminus of SANS lead to the loos of SANS binding to IFT-B molecules. Our findings provide direct evidence for a molecular link between the ciliary USH protein network and the IFT transport module in primary cilia.

## Introduction

The human Usher syndrome (USH) is a complex autosomal recessive disease, characterized by combined vision and hearing loss, accompanied by vestibular dysfunction (Davenport and Omenn, 1977; Wolfrum, 2011; Mathur and Yang, 2015). Clinically, three types of USH (USH1, USH2, and USH3) are distinguished, depending on the onset of vision loss in form of Retinitis pigmentosa (RP), the severity and progression of hearing impairment and the occurrence of vestibular dysfunction. The 10 identified USH genes encode for proteins of diverse protein families, such as the motor protein myosin VIIa (USH1B), the Ca^2+^-integrin binding protein CIB2 (USH1H), the 4-transmembrane protein clarin-1 (USH3A), the single trans-transmembrane protein Usherin/USH2A, the USH cadherins, protocadherin 15 (USH1F) and cadherin 23 (USH1D), the adhesion GPCR VLGR1 (USH2C), and scaffold proteins, namely harmonin (USH1C), whirlin (USH2D) and SANS (scaffold protein containing ankyrin repeats and SAM domain, USH1G) that organize a common USH protein interactome (Wolfrum, 2011; Mathur and Yang, 2015). The decipherment of the protein networks related to USH has strongly contributed to the understanding the pathophysiology of the USH disease in both the inner ear and the retina (El-Amraoui and Petit, 2005; Wolfrum, 2011).

**FIGURE 1:**
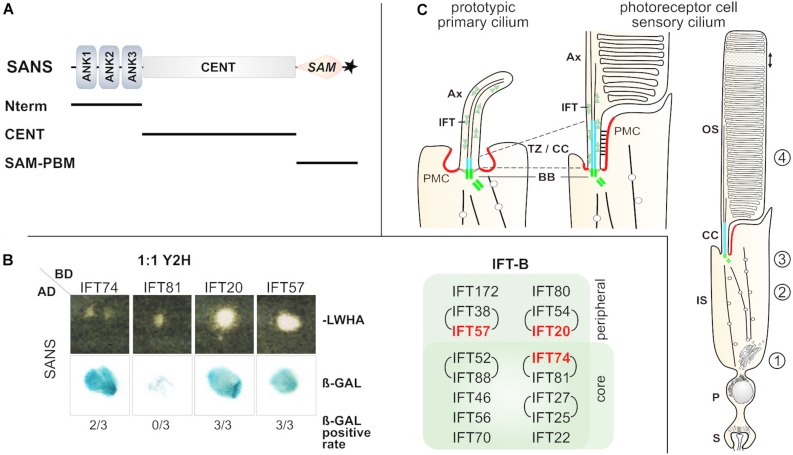
SANS-IFT binary interactions were identified via 1:1 Yeast-two-hybrid. **(A)** Scheme of domain composition of human SANS protein. SANS has 461 aa and a molecular weight of 51 kDa and is composed of the following domains: 3 ANK, ankyrin repeats in the SANS_Nterm; CENT, central domain; SAM, sterile alpha motif; ^∗^PBM, PDZ binding motif. SANS deletion constructs, which were used for GFP-Traps are indicated below by black lines. **(B)** 1:1 Y2H assay. Representative yeast colonies on –LWHA selection media and positive ß-galactosidase assay revealed binding of SANS full length to several IFT-B proteins. For all IFTs three Y2H assays were performed. The rate of ß-GAL positive tests is indicated below, e.g., 2 out of 3 for IFT74 (2/3). Positive hits are highlighted in the IFT-B composition overview in red. **(C)** Schemes of a prototypic cilium and a photoreceptor cilium. Both share homolog structures and common ciliary transport modules: the outer segment (OS) of photoreceptors resembles the ciliary shaft with axoneme (Ax) of the prototypic cilium, and the connecting cilium corresponds to the transition zone (TZ, blue). Intracellular transport for the delivery of ciliary proteins to the outer segment is organized in multiple modules: cargo sorting and vesicle budding at the Golgi apparatus (1), microtubule guided vectorial transport through the inner segment via cytoplasmic dynein (2), vesicle fusion with the periciliary membrane complex (PCM, red) and cargo reloading/assembly at the ciliary base (3) and finally, intra-ciliary transport along the CC/TZ and the Ax (4), mediated by intraflagellar transport (IFT) complexes (IFT-B/kinesin II or IFT-A/dynein-2) or by myosin VIIa, respectively. BB, basal body, green; P, perikaryon, S, synapse.

In the present study we focus on the USH1G protein SANS (scaffold protein containing ankyrin repeats and SAM domain). SANS is composed of three domains, the N-terminal (N-term) domain contains three ankyrin-repeats, the central domain (CENT) and a C-terminal domain which includes a SAM (sterile alpha motif) followed by a class I PBM (PDZ-binding motif) (Figure 1A; Kikkawa et al., 2003; Weil et al., 2003). The CENT domain has been identified as the major interaction domain in the SANS molecule. Yeast-2-hybrid screens and binary protein-protein interaction assays revealed more than 50 putative and validated binding proteins which bind to the CENT domain (Adato et al., 2005; Maerker et al., 2008; Overlack et al., 2011; Sorusch et al., 2014, 2017; Tebbe et al., in preparation; Yildirim et al, in preparation). This long list also includes the two USH proteins, myosin VIIa (USH1B) and whirlin (USH2D) (Adato et al., 2005; Maerker et al., 2008; Sorusch et al., 2017). Two other USH proteins, harmonin, whirlin and the genetic modifier of USH PDZD7 bind with their PDZ domains to the C-terminal PBM (Adato et al., 2005; Maerker et al., 2008; Schneider et al., 2009; Sorusch et al., 2017) interacts the MAGUK protein MAGI2 to an internal PBM in the SAM domain (Bauss et al., 2014). In summary this data qualifies SANS as one of the key organizers of the protein networks related to USH (Maerker et al., 2008; van Wijk et al., 2009; Yang et al., 2010; Zallocchi et al., 2010; Overlack et al., 2011; Bauss et al., 2014; Sorusch et al., 2017).

In the inner ear SANS is part of the upper tip-link density in the mechanosensing stereovilli (=stereocilia) of hair cells, regulating the dynamics of their actin filament core and the mechanotransduction process (Caberlotto et al., 2011; Grati and Kachar, 2011). In addition, SANS associates with the microtubule tracks at the base of the kinocilia of hair cells (Adato et al., 2005). In photoreceptor cells, SANS is related to the microtubule-based intracellular transport machinery (Maerker et al., 2008; Zallocchi et al., 2010; Overlack et al., 2011; Papal et al., 2013), which mediates the targeting of transport vesicles containing ciliary cargo to the base of the photoreceptor cilium (Maerker et al., 2008; Bauss et al., 2014; Sorusch et al., 2017). At the ciliary base, SANS is part of the ternary SANS-USH2A-whirlin protein complex, which is thought to participate at the handover of ciliary cargo from the intracellular transport to the ciliary or intraflagellar transport (IFT) within the photoreceptor cilium (Maerker et al., 2008; Sorusch et al., 2017). Pathogenic mutations in SANS severely disrupted the formation of this USH protein complex (Sorusch et al., 2017). Moreover, the dynamic interaction of SANS with MAGI2 controls receptor-mediated endocytoses in the periciliary region of primary cilia and photoreceptor cilia (Bauss et al., 2014).

The association of SANS and other USH proteins with primary cilia as well as their role in ciliogenesis and ciliary maintenance manifested the USH as a ciliopathy (Bauss et al., 2014; Bujakowska et al., 2017; May-Simera et al., 2017). This group of disorders is characteristically caused by defects in the formation, function or maintenance of cilia (Liu et al., 2010; Sorusch et al., 2014; Wheway et al., 2014). In vertebrate photoreceptor cells the photo-sensitive outer segment resembles a highly modified ciliary shaft of a primary cilium (Figure 1C; Roepman and Wolfrum, 2007; May-Simera et al., 2017). The outer segment of rod photoreceptor cells is characterized by densely packed internal membrane disks which contain the visual pigment rhodopsin and all other components of the photo-transduction cascade. The photoreceptor cilium processes a rudimental ciliary axoneme but an elongated transition zone, named connecting cilium. The USH1B protein myosin VIIa is a prominent component of the connecting cilium and there is evidence that myosin VIIa participates in the anterograde ciliary transport of rhodopsin (Liu et al., 1997, 1999; Wolfrum and Schmitt, 2000). Given that SANS also localizes to the connecting cilium and physically interacts with the myosin VIIa tail both USH1 proteins cooperate in photoreceptor cilia (Adato et al., 2005).

Nevertheless, IFT system is undoubtedly the most common transport machinery in primary cilia (Pedersen and Rosenbaum, 2008; Taschner and Lorentzen, 2016) and constituted in photoreceptor cilia (Sedmak and Wolfrum, 2010; Keady et al., 2011; Zhao and Malicki, 2011). In IFT, IFT particles are the principle transport unites for the bidirectional transport of ciliary cargo. IFT particles are assembled at the ciliary base compartment and consist of two sub-complexes, IFT-A and IFT-B, each are composed of several individual IFT proteins. Mutations in several IFT genes has been implicated in different ciliopathies. While IFT-A is linked to the retrograde motor dynein-1b (dynein-2 in mammals), the anterograde motor kinesin-2 is coupled to IFT-B. The IFT-B proteins assemble into two sub-complexes, the “core” IFT-B1 complex (IFT88/81/74/70/52/46/27/25/22) and the “peripheral” IFT-B2 complex (IFT172/80/57/54/38/20) (Figure 1B; Taschner and Lorentzen, 2016). Although, both the USH-protein related ciliary transport and the IFT system have been analyzed for a long time, no molecular link between them has yet been found yet. Exactly on this “missing link” we focused in our present study. We aimed to identify interactions of the USH1G protein SANS with the IFT-B complex proteins by applying complementary affinity capture approaches. In the present study we describe mutual interactions revealing first molecular linkages between USH protein networks related to the human USH and the IFT system.

## Results

### Yeast-Two-Hybrid (Y2H) Binding Assays Demonstrate Putative Direct Binding of SANS to IFT Complex B Molecules

We have previously demonstrated that USH1G protein SANS acts in transport modules of primary cilia of cultured cells and retinal photoreceptors (Papal et al., 2013; Bauss et al., 2014). Here, we aimed to test the interaction of SANS with the IFT-B complex. In a previous study, the N-terminal ankyrin repeats of SANS exhibit auto-catalytic activity in Y2H screenings (Maerker et al., 2008; Overlack et al., 2008). To circumvent this issue, we applied reciprocal 1:1 Y2H assays with SANS full length (Figure 1A). For this, we transfected yeast cells with either SANS full length fused to the activation domain (pAD) or full length IFT molecules fused to the DNA-binding domain (pBD) of the *GAL4* reporter gene. After mating, positive clones were identified by yeast growth on selection media and positive interactions were visualized by staining for β-galactosidase activity (Maerker et al., 2008). The galactosidase reporter activity revealed the binding of SANS to IFT proteins, namely IFT74, IFT20, and IFT57 in the Y2H assay (Figure 1B). In contrast, there was no interaction between SANS and IFT81 detected (Figure 1B). The most frequent interaction (in three out of three experiments) has been observed with IFT20 and IFT57, both of which are components of the peripheral IFT-B2 complex (Figure 1B; Lucker et al., 2005; Taschner et al., 2014; Taschner and Lorentzen, 2016). For IFT74 which is a component of the IFT-B1 core complex (Taschner and Lorentzen, 2016) we found interactions to SANS in two out of three experiments (Figure 1B). During the formation of the IFT-B complex, the B1 and B2 sub-complexes assemble via the interaction of IFT57 (IFT-B2) and IFT52 (IFT-B1) (Taschner and Lorentzen, 2016). Thus, we included IFT52 in subsequent analyses. Although, we observed binding of the IFT-A complex IFT140 to SANS in two out of three 1:1 Y2H assays (data not shown), we decided to focus on SANS-IFT-B complex interactions and did not to further evaluate the interaction of SANS to IFT-A complex molecules.

### *In vitro* GFP-Trap Pull Down Assays Confirm Interaction of SANS With IFT57 and IFT52

**FIGURE 2:**
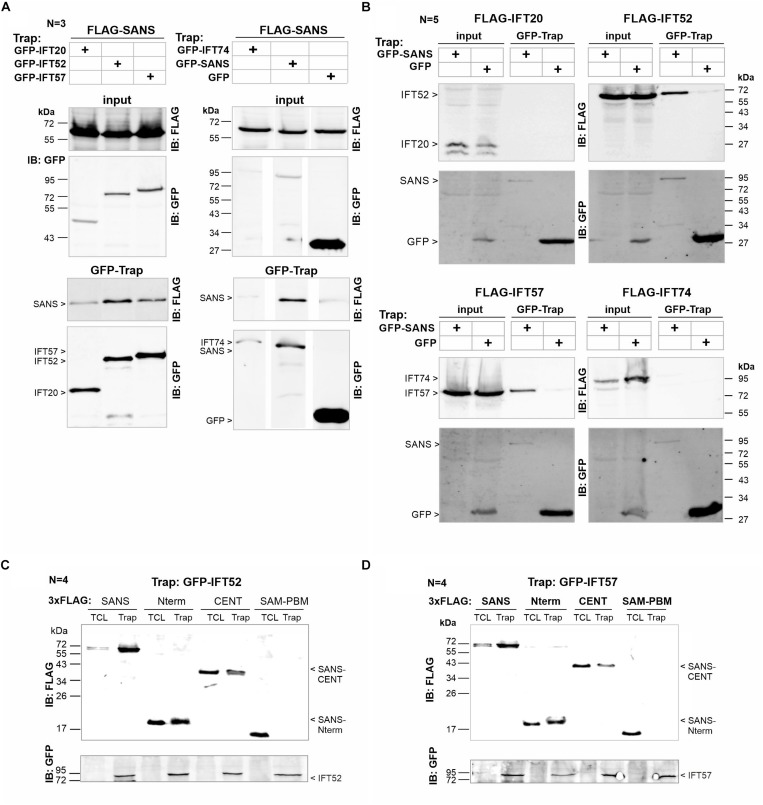
Bidirectional confirmation of SANS’ interaction with IFT20, IFT52, and IFT57 *in vitro*. **(A)** Representative Western blot analysis of GFP-Trap of FLAG-SANS full-length by GFP-tagged IFT20, IFT52, IFT57, and IFT74, immobilized on GFP-Trap beads of three independent experiments. Controls: GFP-SANS (positive) and GFP alone (negative). FLAG-SANS was precipitated by IFT20, IFT52, IFT57 (upper panel), and GFP-SANS, but not by IFT74 or GFP alone (lower panel). **(B)** Representative Western bolt analysis of reciprocal GFP-Trap of FLAG-IFT20, FLAG-IFT52, FLAG-IFT57, and FLAG-IFT74 by GFP-SANS and GFP (control), respectively of five independent experiments. FLAG-tagged IFT52 and IFT57 were precipitated by GFP-SANS, immobilized on GFP-Trap beads, but not by GFP alone. No interaction was detected with IFT20 or IFT74. **(C,D)** Representative Western blot analyses of GFP-Trap of FLAG-SANS full length, Nterm, CENT, and SAM-PBM by GFP-tagged IFT52 and IFT5, respectively, immobilized on GFP-Trap beads. FLAG-SANS Nterm and FLAG-SANS-CENT were precipitated by both, GFP-IFT52 and GFP-IFT57.

Next, we aimed to validate the putative binding to SANS by independent interaction assays *in vitro*. For this, we co-expressed FLAG-SANS with GFP-tagged IFTs or in a vice versa experiment used GFP-SANS and FLAG-tagged IFTs in HEK293T cells. Bound to GFP-Trap beads, GFP-tagged IFT20, IFT52, and IFT57 were precipitating FLAG-SANS from HEK293T cell lysate (Figure 2A, upper panel), whereas GFP-IFT74 and GFP alone did not interact (Figure 2A, lower panel). When GFP alone or GFP-SANS, respectively, were immobilized on GFP-Trap beads, we detected the specific interaction of GFP-SANS with FLAG-IFT52 and FLAG-IFT57 (Figure 2B). In contrast, neither FLAG-IFT20 nor FLAG-IFT74 were precipitated by GFP-SANS (Figure 2B). No unspecific binding of FLAG-tagged IFT proteins to GFP alone was observed. Together, the results of our bidirectional GFP-Trap confirmed the direct molecular interaction of SANS with IFT20 and additionally demonstrated the interaction of SANS with IFT52 and IFT57, respectively. In contrast, IFT74 did not bind to SANS in *in vitro* GFP-Trap pull down assays.

### Identification of the N-Terminal Ankyrin Repeat Domain of SANS as the Major Binding Domain for IFT Molecules

We next extended our biochemical analysis by pointing to the IFT-binding domain in SANS. For this, we co-expressed GFP-IFT52 and GFP-IFT57 with 3xFLAG-tagged SANS constructs, namely SANS-Nterm, containing ankyrin repeats, SANS-CENT and C-terminal SANS-SAM-PBM, respectively (Figures 1A, 2C,D). In the subsequent GFP-Trap pulldown both, GFP-IFT52 and GFP-IFT57, were recovered by the SANS-Nterm in two out of four independent experiments. In addition, weaker binding between both GFP-tagged IFTs and SANS-CENT were observed in all four experiments performed (Figures 2C,D).

### Membrane Targeting Assays Confirmed SANS-IFT-B Protein Interaction in the Environment of the Eukaryotic Cell

**FIGURE 3:**
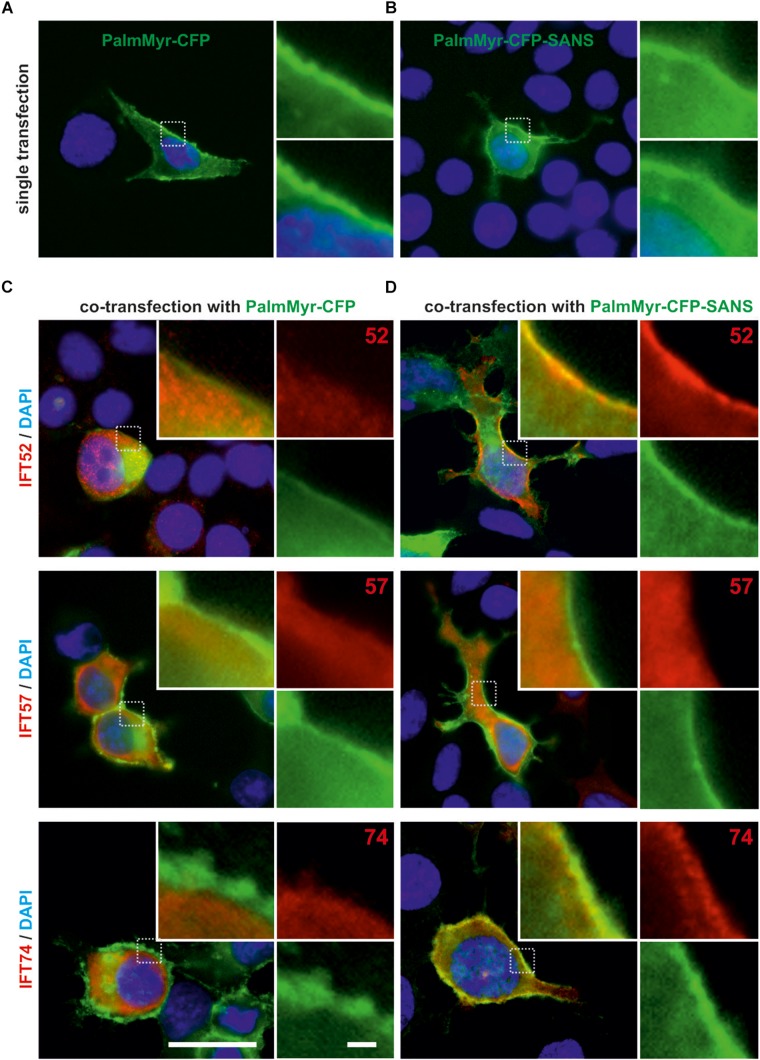
Membrane targeting assay in HEK293T cells revealed *in vivo* translocation of IFT52 and IFT74 by PalmMyr-CFP-SANS. **(A,B)** Fluorescence microscopic analysis of HEK293T cells, singly transfected with MyrPalm-CFP or MyrPalm-CFP. MyrPalm-CFP **(A)** and MyrPalm-CFP-SANS (both in green) **(B)**, respectively, accumulate at the plasma membrane of single-transfected HEK293T cells. **(C,D)** Fluorescence microscopic analysis of HEK293T cells, co-transfected with FLAG-tagged IFT20, IFT52, IFT57 or 74, and with MyrPalm-CFP **(C)** or MyrPalm-CFP-SANS **(D)**, respectively. In the control, no co-localization of FLAG-IFT proteins with MyrPalm-CFP alone was observed. In contrast, FLAG-IFT52 and FLAG-IFT74 co-localized with MyrPalm-CFP-SANS at the plasma membrane, whereas FLAG-IFT20 and FLAG-IFT57 did not. Blue, DAPI staining of nuclear DNA. Images are representative for co-transfected cells from two independent experiments. Scale bars: 25 μm; 2.5 μm in zoomed squares.

Next we tested the interaction of SANS with the IFT-B components in the environment of mammalian cells. For this, we applied membrane targeting assays in HEK293T cells. We expressed CFP-tagged SANS fused to a palmitoylation-myristoylation (PalmMyr) membrane anchor. Immunofluorescence microscopy demonstrated that as expected, PalmMyr-CFP and PalmMyr-CFP-SANS fusion proteins were targeted to the plasma membrane (Figures 3A,B). Next, we did co-transfection of FLAG-tagged IFT proteins with PalmMyr-CFP-SANS and PalmMyr-CFP alone, respectively (Figures 3C,D). Immunofluorescence microscopy of anti-FLAG demonstrated that in cells co-expressing PalmMyr-CFP and FLAG-IFT proteins the IFT proteins were evenly distributed in the cytoplasm of the co-transfected cells of negative controls (Figure 3C). In contrast, in cells co-expressing PalmMyr-CFP-SANS and FLAG-IFT52 or FLAG-IFT74, respectively, both IFTs were enriched at the plasma membrane (Figure 3D). In the case of co-expression of PalmMyr-CFP-SANS and FLAG-IFT57 we did not observe a recruitment of FLAG-tagged IFT to the plasma membrane.

The inconsistences in binding of IFT74 and IFT57 to SANS which observed between the two different binding assays, the *in vitro* binding assays and the membrane targeting assays are difficult to explain, but might be due to the different experimental conditions. Both the buffer systems used in the *in vitro* assays and physiological status of proteins in the cells for the membrane targeting assay may differentially affect the molecular structure and thereby the binding affinity of the divers IFTs to SANS.

### Co-localization of SANS and Interacting IFT Molecules in Retinal Photoreceptor Cells

**FIGURE 4:**
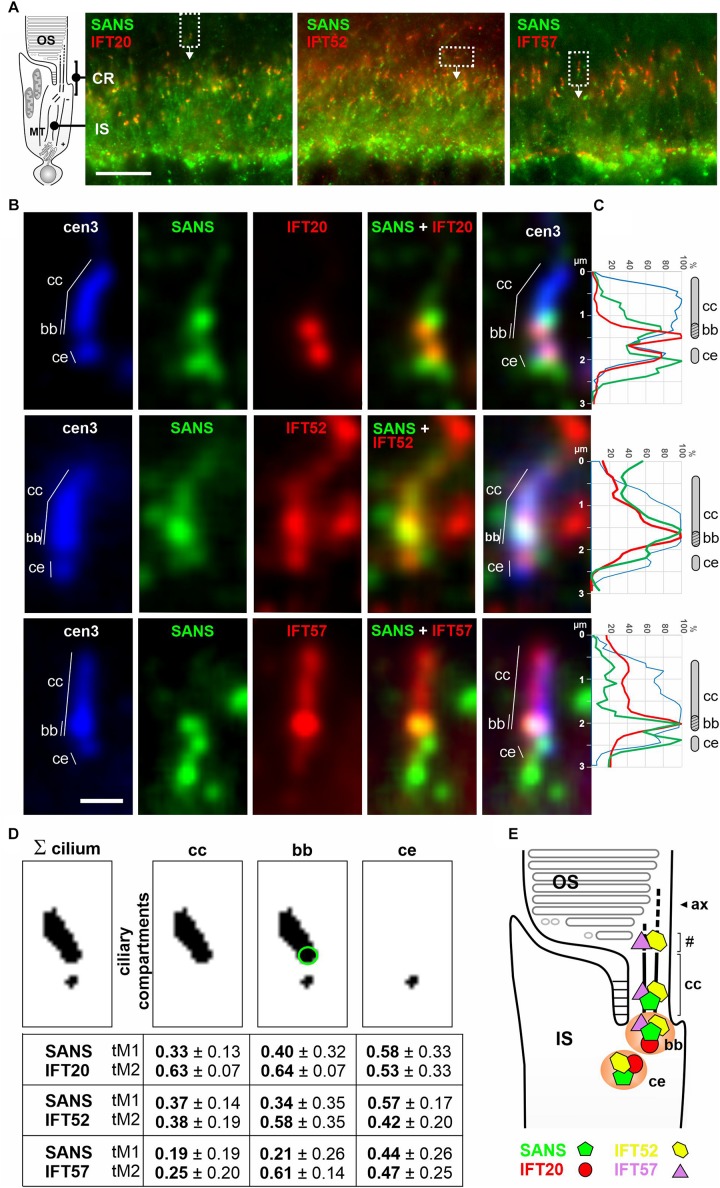
Subcellular localization of SANS and IFT-B proteins in the ciliary region of murine photoreceptor cells. **(A)** Indirect immunofluorescence triple labeling of SANS and with IFT20, IFT52, and IFT57 of photoreceptor layers in longitudinal cryosections of murine wild type retinas co-stained with anti-centrin3 (cen3), a marker for the connecting cilium (cc), basal body (bb) (mother centriole), and the adjacent (daughter) centriole (ce) of photoreceptor cells. CR, ciliary region; OS, photosensitive outer segments; the IS, inner segment; MT, microtubule tracks through the IS. **(B)** High magnification of photoreceptor cilia from areas indicated by rectangles in **(A)**. **(C)** Intensity plots of the immunofluorescence of the photoreceptor cilia shown in **(B)**. **(D)** Measurements of the Manders’ Colocalization Coefficients (MCC) of subciliary compartments of photoreceptor cilia, namely the cc (highlighted by green circle) and ce. Numbers from 0 to 1 indicate increasing co-localization of molecules M1 (SANS) and M2 (IFT). **(E)** Schematic illustration of the localization of SANS (green), IFT20 (red), IFT52 (yellow), and IFT57 (purple) in photoreceptor cilia. Partial co-localization of SANS with the IFT-B molecules at the ciliary base is indicated by orange color. # indicates OS base where disk neogenesis takes place. Ax, axoneme. Scale bar: 10 μm **(A)**; 1 μm **(B)**.

We have previously shown that SANS is predominately localized at the ciliary base but also found in the connecting cilium (=transition zone) of photoreceptor cilia (Maerker et al., 2008; Bauss et al., 2014; Sorusch et al., 2017). In our previous analysis of the spatial distribution of IFT proteins in photoreceptor cells, we also predominately found IFT molecules at the base of the photoreceptor cilium (Sedmak and Wolfrum, 2010). While IFT20 was restricted to the ciliary base, IFT52 and IFT57 were also localized to the connecting cilium and additionally accumulated in a compartment proximal to the connecting cilium, known as the site for outer segment disk neogenesis at the base of the photoreceptor outer segment (Sedmak and Wolfrum, 2010). These findings prompted us to test, whether SANS and the IFT-B proteins indeed co-localize in the photoreceptor cilium. For this, we triple immuno-labeled longitudinal cryosections across the murine retina with anti-centrin 3, a reliable molecular marker for the connecting cilium and the basal body of photoreceptor cells (Trojan et al., 2008), anti-SANS and antibodies against IFT20, IFT52, or IFT57, respectively (Figure 4). Confocal immunofluorescence microscopy affirmed the spatial distribution of the investigated molecules previously described: a localization of SANS predominantly at the ciliary base, the localization of all three IFT molecules at the ciliary base, IFT52 and IFT57 additionally in the connecting cilium of photoreceptor cells (Figures 4A,B; Maerker et al., 2008; Sedmak and Wolfrum, 2010; Bauss et al., 2014; Sorusch et al., 2017). The quantification of the immunostaining in photoreceptor cilia was accessed by intensity plots of the immunofluorescence of single photoreceptor cilia (Figure 4C) and measured via Manders’ Colocalization Coefficients (MCC) (Figure 4D). Our analyses reveal partial co-localization of SANS with the IFT-B molecules at the ciliary base, namely the basal body (mother centriole) and the adjacent centriole (daughter centriole) of photoreceptor cilia. Thus, SANS may interact with IFT52 and IFT57 at the ciliary base and the connecting cilium, while the interaction of SANS with IFT20 may be restricted to the ciliary base.

### Analysis of IFT-B Protein Expression in Photoreceptor Cells of SANS Knock Out (ko) Mice

**FIGURE 5:**
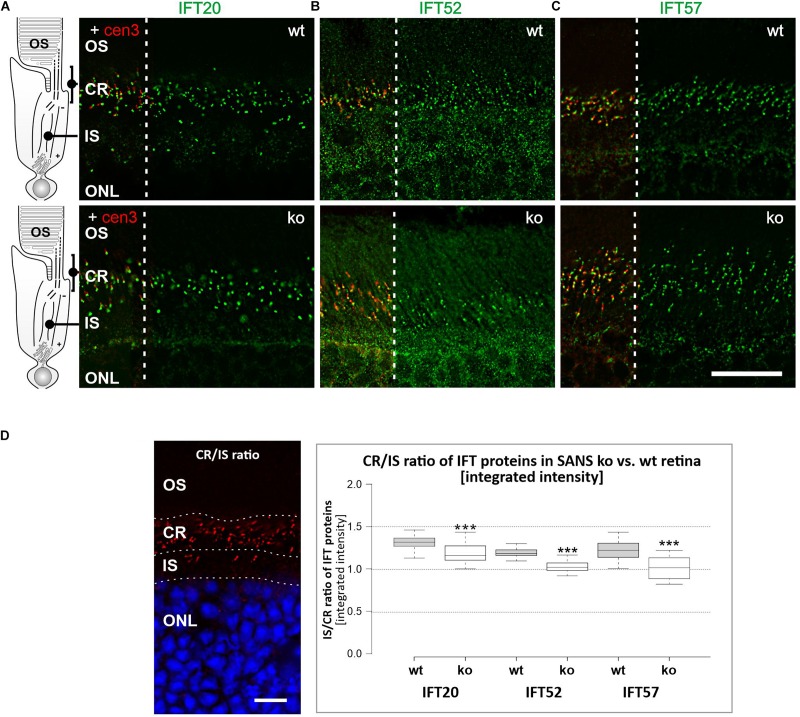
Analysis of IFT-B protein expression in photoreceptor cells of SANS knock out (ko) mice. Immunofluorescence labelling of the photoreceptor layer of wild type mice (upper panel) and SANS ko mice (lower panel) by anti-IFT-B molecules, IFT20 **(A)**, IFT52 **(B)**, and IFT57 **(C)**, respectively, counterstained for nuclear DNA by DAPI. Scale bar: 10 μm. **(D)** BoxPlot analysis of fluorescence intensities of anti-IFT-B staining in the ciliary region (CR) versus inner segment region (IS) in wild type and SANS ko mice. The presence of all three IFT-B molecules in the ciliary region is significantly lower in SANS ko mouse photoreceptor cells.

Next we aimed to study whether the absence of SANS from primary cilia effects the spatial distribution of IFT-B molecules in the primary sensory cilia of photoreceptor cells. For this we stained longitudinal cryosections through retinas of wild type and *Ush1g*^–/–^ C57BL/cJ (SANS ko) mice (Caberlotto et al., 2011) and subsequently compared the intensities of the immunofluorescence in the ciliary region and the non-ciliary inner segment of the photoreceptor layer in these retinas (Figures 5A,B). The BoxPlot analyses of the fluorescence staining reveled significant lower intensities in the ciliary region of photoreceptor cells of SANS ko mice when compared to wild type mice (Figure 5C).

### SANS Mutations Disturbed Interactions of SANS With IFT52 and IFT57

Next, we validated the effects of pathogenic mutations in the N-terminal domain of human *SANS* on the protein-protein interaction of SANS with IFT52 and IFT57 in two independent experiments. For this, introduced p.Leu48Pro and p.Met104Val (Maria Oonk et al., 2015) into human *SANS* by site-directed mutagenesis into the FLAG-tagged SANS (Supplementary Figure S1A). Subsequently, we co-expressed FLAG-tagged wild type or mutated SANS, respectively, with GFP-tagged IFTs in HEK293T cells for GFP-Trap assays (Supplementary Figure S1B). Quantification of Western blot bands showed that the recovery of mutated SANS polypeptides was very low (Supplementary Figure S1C).

## Discussion

In this present study, we demonstrate the interaction between the USH type 1 protein SANS (USH1G) and proteins of the anterograde intraflagellar transport (IFT-B). Complementary protein-protein interaction assays revealed that SANS can interact with IFT20, IFT52, IFT57, and IFT74.

Others and ourselves have previously identified numerous proteins interacting with the USH1G protein SANS (Adato et al., 2005; Maerker et al., 2008; Overlack et al., 2011; Bauss et al., 2014; Sorusch et al., 2017). Most interacting molecules bind to the CENT domain which has been shown to mediate the homodimerization of SANS (Maerker et al., 2008; Overlack et al., 2008; Sorusch et al., 2014, 2017; Tebbe et al., in preparation; Yildirim et al., in preparation). In addition, PDZ-domain containing scaffold proteins bind to the PDZ-binding motif (PBM) at the C-terminus (Adato et al., 2005; Maerker et al., 2008; Sorusch et al., 2017) or to an internal PBM in the SAM domain of the C-terminal domain (Bauss et al., 2014). Here, for the first time, we identified molecules binding to N-terminal domain of SANS which contain at least three predicted ankyrin (ANK) repeats (Kikkawa et al., 2003). In other proteins ANK repeats are potent interaction sites underlying scaffold functions (reviewed in: (Li et al., 2006; Islam et al., 2018), therefore the presence of ANK repeats led to SANS being predicted to act as a scaffold protein (Kikkawa et al., 2003). Present *in vitro* interaction assays demonstrate that IFT52 and IFT57 bind directly to the N-terminal domain of SANS, containing these ANK repeats (Figures 2C,D). A binding of IFT52 and IFT57 to the N-terminus of SANS is also in line with the disturbed binding of SANS molecules containing pathological mutations in the N-terminus observed in our preliminary GFP-Trap assays. Latter GFP-Traps also show minor binding of both, IFT52 and IFT57, to SANS_CENT (Figures 2C,D). Since SANS_CENT peptides can dimerize with intrinsic full length SANS (Adato et al., 2005; Sorusch et al., 2017), SANS_CENT may co-precipitate with intrinsic full length SANS bound to the GFP-tagged IFTs in GFP-Traps. Nevertheless, a binding of IFTs to the SANS_CENT with a weaker affinity cannot be excluded and remains to be solved in future studies.

In addition to the binding of SANS to IFT52 and IFT57 we observed interactions of SANS with IFT20 and IFT74 (Figures 1B, 2A). However, it is notable that the data were not consistent in the different protein-protein interaction assays. While the interaction of SANS with IFT52 was most consistent and reproducible, IFT20 did not bind and IFT74 showed only a very weak binding to SANS in the *in vitro* GFP-Trap assays (Figures 2A,B). IFT57 and IFT20 were not recruited by PalmMyr-SANS in the membrane targeting assay (Figure 3). As already mentioned above this might be due to the different experimental conditions in the different assays. Nevertheless, our findings support the hypothesis that SANS associates with the anterograde transport machinery of the IFT-B complex by interacting with both of its subcomplexes, the peripheral IFT-B1 and the core IFT-B2. This hypothesis is further strengthened by the co-localization of SANS and IFT-B molecules in the cilium of mouse photoreceptor cilia. Latter data are in line with the findings from the single localization analyses of SANS and IFT-B proteins, respectively, in photoreceptor cells (Pazour et al., 2002; Maerker et al., 2008; Overlack et al., 2008; Sedmak and Wolfrum, 2010; Sorusch et al., 2017).

Thus, what is the role of the interaction between SANS and IFT-B components in cilia physiology? There is no doubt that transport along the cilium is based on the IFT machinery and that the IFT-B complex in IFT particles is responsible for the anterograde transport of ciliary cargo along the ciliary microtubules to the ciliary tip (Pedersen and Rosenbaum, 2008). Nevertheless, others and we have shown that an alternative transport machinery for ciliary cargo such as opsin powered by the myosin VIIa motor protein exists in the sensory primary cilia of photoreceptor cells (Liu et al., 1997, 1999; Wolfrum and Schmitt, 2000). Although previous proximity ligation assays (PLAs) have indicated that the USH proteins Cdh23, harmonin, and myosin VIIa are in close proximity to IFT88 in zebrafish hair cells, so far, no direct interaction between myosin VIIa and any molecules of the IFT machinery has been found (Kussel-Andermann et al., 2000; Blanco-Sanchez et al., 2014). However, mutations in both genes *MYO7A* and *SANS* cause USH type 1, namely subtypes USH1B and USH1G, respectively (Wolfrum, 2011). More importantly the proteins encoded by both USH1 genes are part of the common interactome related to the USH and physically interact with each other (Adato et al., 2005; Wolfrum, 2011). Therefore, SANS may serve as a molecular linker between both anterograde intraciliary transport systems in photoreceptor cells. This hypothesis is supported by the co-localization of SANS with myosin VIIa and IFT-B molecules in the photoreceptor cilium and by its direct binding to myosin VIIa and to a set of IFT-B proteins. The molecular function of SANS as a “connector” depends on two different domains, namely SANS_CENT and SANS_Nterm for binding to myosin VIIa or to IFT-B molecules, respectively. This is in line with previously described linker proteins such as dystonin or pectins which can bridge intermediate filaments, actin filaments and microtubules and contain different binding domains for each cytoskeletal element (Fuchs and Cleveland, 1998). IFT molecules are ubiquitously expressed in ciliated cells and the presence of myosin VIIa and SANS is also not restricted to photoreceptor cells, but found in other primary ciliated cells, too (e.g., Wolfrum et al., 1998; Bauss et al., 2014). Therefore, SANS’s linker role for the anterograde ciliary transport machinery might be common for primary cilia. Nevertheless, since we also observed in an none-validated initial 1:1 Y2H assay the binding of SANS to the IFT-A component IFT140 and our previous studies suggests partial co-localization of SANS with IFT140 in photoreceptor cilia (for IFT140 see, Sedmak and Wolfrum, 2010; for SANS see, e.g., Maerker et al., 2008; Sorusch et al., 2017 and present study, Figure 4) we cannot exclude that SANS is related to the IFT-A complex responsible for the retrograde transport in cilia.

In the present study, co-labeling experiments revealed that SANS co-localizes with IFT20, IFT52 and IFT57 at the base of sensory primary cilium of photoreceptor cells. Single labeling studies of SANS and IFTs suggest that this is also the case for other primary cilia (Pazour et al., 2002; Sedmak and Wolfrum, 2010; Bauss et al., 2014). In photoreceptor cells, transmembrane proteins, such as rhodopsin, are transported as cargo in membranous vesicles along microtubules by cytoplasmic dynein, from the Golgi apparatus across the inner segment to the periciliary compartment at the base of the cilium (Tai et al., 1999; Papermaster, 2002). We have previously shown that SANS is associated with these cargo vesicles (Papal et al., 2013). At the ciliary base the transport vesicles dock and fuse with periciliary membrane and by this the transmembrane cargo molecules are incorporated into the target membrane for their ciliary delivery (Papermaster, 2002; Maerker et al., 2008). There is growing evidence that SANS plays an important role in this process which implements the handover of cargos from the minus-end-directed cytoplasmic dynein transport module to the kinesin-II and/or myosin VIIa powered ciliary transport (Papermaster, 2002; Maerker et al., 2008; Bauss et al., 2014).

In the periciliary compartment at the ciliary base, the IFT-B complex assembles and is integrated into IFT-particles as an adaptor between the anterograde heterotrimeric kinesin motor proteins and the ciliary cargo (Sedmak and Wolfrum, 2010; Lechtreck, 2015). IFT57 modulates the incorporation of kinesin II to the IFT-B complex (Krock and Perkins, 2008). Moreover, IFT52 and IFT57 provide a physical link between the peripheral IFT-B2 subcomplex and the IFT-B core (IFT-B1) by direct interaction (Taschner and Lorentzen, 2016). Given that IFT52 and IFT57 are essential components for the assembly of architecture of the IFT-B protein complex, it is, in our opinion a lucrative hypothesis that the molecular interaction between the IFT-B molecules and SANS may control the transport of IFT-B molecules to the ciliary base, the assembly of the IFT-B subcomplexes at the ciliary base and its delivery into the ciliary shaft. This is in line with the disturbed presence of IFT-B molecules in the SANS deficient photoreceptor cilia of the SANS ko mice. Our preliminary data on the effect of pathogenic mutations in SANS N-terminus on the interaction of SANS with IFT-B molecules indicate that these may also disturb the IFT-B system which may contribute to the pathophysiology in USH1G.

## Conclusion

In summary, our studies provide novel insight into the functional relationship between USH1G protein SANS and the ciliary transport machinery IFT. We uncovered the direct physical interaction between SANS and particles of the IFT-B complex. These interactions provide the first direct molecular link between the ciliary/periciliary USH protein networks and the IFT machinery. Moreover, our findings facilitate a function of SANS and SANS associated USH protein complexes in microtubule- based transport processes of ciliary cargoes. Furthermore mutations in *SANS* alter SANS-IFT-B interaction which may consecutively lead to ciliary transport defects underlying the pathomechanisms of USH type 1G.

## Materials and Methods

### Antibodies and Fluorescent Dyes

Polyclonal SANS antibodies generated against a murine fragment (amino acids 1–46), raised in rabbit, and guinea pig anti-SANS_CENT (against human amino acid 339–384). Monoclonal antibodies against Centrin3 were used as molecular markers of the connecting cilium and the basal body of photoreceptor cells (Trojan et al., 2008). Polyclonal rabbit antibodies raised against IFT20, IFT52, and IFT57, respectively, were kindly provided by G. J. Pazour (University of Massachusetts Medical School, Worcester, MA, United States). Commercially available antibodies were used as followed: anti-FLAG (SAB1404762) from Sigma-Aldrich (Hamburg, Germany). Secondary antibodies were conjugated to Alexa488, Alexa568, or Alexa 647, purchased from Molecular Probes (Life Technologies, Darmstadt, Germany) or from Rockland Inc. (Gilbertsville, PA, United States). Nuclear DNA was stained with DAPI (4′,6-diamidino-2-phenylindole, 1 mg/ml; Sigma-Aldrich).

### DNA Clones and Constructs

Cloning of cDNA for expression of SANS was previously described (Maerker et al., 2008). The numbers of the given amino acids are according to the following Genbank entries: SANS (human, NP_775748), IFT20 (NM_001267774), IFT52 (NM_001323580), IFT57 (NM_018010), IFT74 (NM_001099221), IFT81 (NM_001143779). IFT cDNA was kindly provided by R. Roepman and E. van Wijk (Nijmegen, Netherlands). For yeast-2-hybrid, anti-GFP nanobody immunoprecipitation [GFP-Trap, beads (ChromoTek, Planegg-Martinsried, Germany)] and membrane targeting assays full length SANS or following deletion constructs of SANS were used: SANS Nterm (aa 2–126), CENT (aa 125–388), SAM-PBM (aa 385–461). cDNA was subcloned into either pCMV-3xFLAG (Invitrogen^TM^), pDest53-GFP or pPalmMyr-CFP (plasmid 14867, Addgene, Cambridge, MA, United States) for expression in HEK293T cells using the Gateway L/R clonase II enzyme mix (Invitrogen^TM^, Karlsruhe, Germany).

### Yeast-2-Hybrid (Y2H)

1:1 yeast-two-hybrid (Y2H) assays were performed as previously described (Overlack et al., 2011). Briefly, full length IFT20, IFT57, IFT74, and IFT81 were fused to the DNA-binding domain (BD) of the GAL4 transcription factor DNA-binding and used as bait, full length human SANS (NCBI: NM_173477) was fused to the DNA-activation domain (AD) of GAL4 transcription factor. In the subsequent 1:1 Y2H, bait and prey were transformed in the yeast strains *PJ694α* and *PJ694A*, respectively. After mating, yeast colonies were selected according to the HIS1, ADE3, LacZ, and MEL1 reporter genes to identify positive interactions. X-ß-gal colorimetric filter lift assay (LacZ reporter gene) was performed to confirm the interaction (Maerker et al., 2008; Overlack et al., 2011).

### Cell Culture

HEK293T (human embryonic kidney cells) were cultured in Dulbecco’s modified Eagle’s medium (DMEM) containing 10% heat-inactivated fetal calf serum (FCS). Cells were transfected with plasmids using GeneJuice^®^ Transfection Reagent (Merck KGaA, Darmstadt, Germany) according to manufacturer’s instructions.

### GFP-Trap Nanobody Immunoprecipitation

GFP polypeptides were immobilized on 25 μl Trap agarose beads (ChromoTek, Planegg-Martinsried, Germany) each and used for co-precipitation assays according to the manufacturer’s protocol. Briefly, cell lysates were obtained from HEK293T cells, co-transfected either with both GFP-tagged SANS and 3xFLAG-IFT20, 52, 57, 74 or with GFP-tagged IFTs and 3xFLAG-tagged SANS or 3xFLAG-tagged mutated SANS. We introduced pathogenic mutations p.Leu48Pro and p.Met104Val (Maria Oonk et al., 2015) into human *SANS* (Figure 5) by using QuikChange Lightning Site-Directed Mutagenesis Kit according to manufacturer’s protocol (Agilent Technologies, Waldbronn, Germany).as previously described (Sorusch et al., 2017). For this, cells were suspended in lysis buffer (10 mM Tris–Cl, pH 7.5, 150 mM NaCl, 0.5 mM EDTA, 0.5% NP-40), spun and the supernatant was diluted to 500 μl in dilution buffer (10 mM Tris–Cl, pH 7.5, 150 mM NaCl, 0.5 mM EDTA). 10% of total cell lysate were separated as input and remaining samples were added to pre-washed beads for 2 h at 4°C under constant shaking. After several washing steps, precipitated protein complexes were eluted with SDS-sample buffer and subjected to SDS-PAGE and Western blot as previously described in Bauss et al. (2014), Sorusch et al. (2017).

### Membrane Targeting Assay

Human SANS was fused to the N-terminal membrane-anchoring peptide and CFP expressed by the PalmMyr-CFP vector (plasmid 14867, Addgene, Cambridge, MA, United States). PalmMyr-CFP-SANS was single- or co-transfected with 3xFLAG-IFT20, 52, 57, 74. Cells were subjected to immunocytochemistry 24 h post-transfection (see also Sorusch et al., 2017). Analysis has been performed on 27 FLAG-IFT20/PalmMyr-CFP-SANS, 14 FLAG-IFT52/PalmMyr-CFP-SANS, 26 FLAG-IFT57/PalmMyr-CFP-SANS, 16 FLAG-IFT74/PalmMyr-CFP-SANS, 13 FLAG-IFT20/PalmMyr-CFP, 9 FLAG-IFT52/PalmMyr-CFP, 32 FLAG-IFT57/PalmMyr-CFP and 20 FLAG-IFT74/PalmMyr-CFP, co-transfected cells from two independent experiments each.

### Immunocytochemistry

Cells were washed in PBS, fixed with 2% PFA, subsequently permeabilized using 0.1% Trion X-100 in PBS, washed 3-times with PBS followed by blocking (0.5% cold-water fish gelatin, 0.1% ovalbumin in PBS) for at least 30 min before primary antibodies were incubated overnight at 4°C. After washing, samples were incubated with secondary antibodies (1:400) and DAPI (1:8,000) for 1 h at room temperature. After washing, cover slips were mounted in Mowiol (Roth, Karlsruhe, Germany).

### Animals and Tissue Dissection

All experiments were carried out in accordance with European Community Council Directives (86/609/EEC) and the ARVO (Association for Research in Vision and Ophthalmology) statement for the use of animals in ophthalmic and visual research. Wild type C57BL/6J mice and *Ush1g*^–/–^ C57BL/cJ mice (Caberlotto et al., 2011) were maintained under a 12 h light-dark cycle, with food and water *ad libitum*. After sacrifice of the animals in CO_2_, eyeballs were dissected for further procedures.

### Immunohistochemistry

Eyes of mice were dissected and eye cups were cryofixed in melting isopentane and cryosectioned as described elsewhere (Wolfrum, 1991). Cryosections were placed on poly-L-lysine-precoated coverslips, incubated with 0.01% Tween 20 PBS, followed by a blocking step for at least 30 min in blocking solution (0.5% cold-water fish gelatin, 0.1% ovalbumin in PBS). Primary antibody incubations were performed over-night at 4°C. Washed cryosections were incubated with secondary antibodies in blocking solution containing DAPI for 1 h at room temperature. After washing, sections were mounted in Mowiol.

### Image Processing

Specimen were analyzed on a Leica DM6000B microscope (Leica, Bensheim, Germany), images were processed with Leica imaging software and ImageJ/Fiji software (Schindelin et al., 2012, 2015).

### Quantification of Immunofluorescence

All image processing and quantification steps have been performed with Fiji/ImageJ. For co-localization analysis we measured the Manders Colocalization Coefficient (Dunn et al., 2011) by applying the ImageJ/Coloc2 plugin on following ciliary regions of interest: the connecting cilium (cc), the basal body (bb) and the centriole (ce). Briefly, a 3–5 z-layer stack containing a centrin-3 labeled cilium was cropped and z-projected. A binary image of the connecting cilium was created by thresholding. Using the particle analyzer and image calculator, the connecting cilium and the centriole were identified (Figure 4D). For analysis of bb, we applied a circular region of interest the size of ce at the base of cc.

For generating fluorescent intensity plot profiles of immunolabeling along photoreceptor cilia, a region of interest was applied on the cilium, using the segmented line tool. For background subtraction, the gray values of each channel were measured in a circular region next to cc, twice the size of ce and subtracted from the values of the according channel.

For the CR/IS ratio of IFT labeling intensity in wildtype (wt) vs. Ush1g^–/–^ C57BL/cJ (*Ush1g*-ko) mice, the integrated density (IntDen) of IFT fluorescence in rectangular regions of interest (ROI, 20.26 × 8.04 μm/length × height) were analyzed. Five z-stacks of wt and *Ush1g*-ko retina, respectively, were analyzed by measuring IntDen of 3–4 ROIs in the region of the inner segment and in 3–4 ROIs in the according ciliary region (see Figure 5), which is defined by positive centrin-3 immunostaining.

## Data Availability Statement

All datasets generated for this study are included in the manuscript/Supplementary Files.

## Ethics Statement

The animal study was reviewed and approved by Landesuntersuchungsamt, Abteilung 2, Referat 23 – Tierseuchenbekämpfung, Tierschutz, tierische Nebenprodukte, Koblenz.

## Author Contributions

BK, NS, and UW designed the experiments. NS and UW wrote the manuscript. BK contributed to Y2H assay experiments. JJ, NS, AY, and WF contributed to the cloning of cDNA constructs and recombinant expression of protein constructs. CS, JJ, WF, and AY contributed to GFP-Trap experiments. JJ and NS contributed to membrane targeting assays. CS and NS contributed to the immunofluorescence analysis on retinal sections. NS performed quantitative analysis of immunofluorescence data.

## Conflict of Interest

The authors declare that the research was conducted in the absence of any commercial or financial relationships that could be construed as a potential conflict of interest.

## Supplementary Material

FIGURE S1 Effects of pathogenic *SANS* mutations on SANS-IFT interaction. **(A)** Localization pathogenic mutations p.Leu48Pro and p.Met104Val in the SANS molecule into human SANS. **(B)** Western blot analysis of GFP-Trap of FLAG-SANS, wild type, p.Leu48Pro and p.Met104Val, respectively, by GFP-tagged IFT52 and IFT57, immobilized on GFP-Trap beads. **(C)** Quantification of the recovery of FLAG-tagged SANS polypeptides by IFT-GFP-Traps indicating that the recovery of mutated SANS protein is drastically reduced.
