# Supplementary figures and images for: SANS (USH1G) Molecularly Links the Human Usher Syndrome Protein Network to the Intraflagellar Transport Module by Direct Binding to IFT-B Proteins
Source: Front Cell Dev Biol. 2019 Oct 4;7:216. doi: 10.3389/fcell.2019.00216 (PMC6787559; doi:10.3389/fcell.2019.00216)

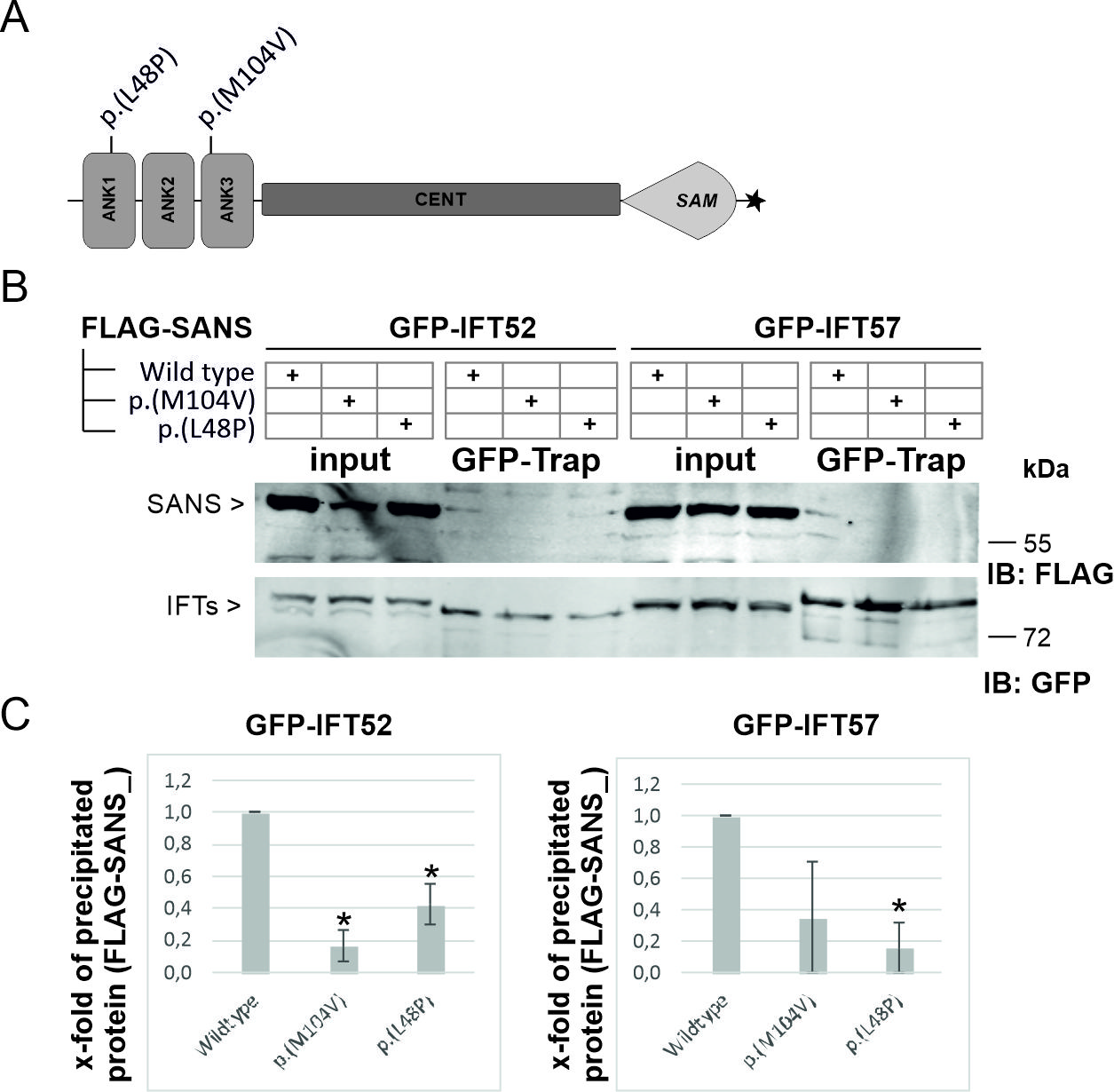

Supplement: FIGURE S1 — Effects of pathogenic SANS mutations on SANS-IFT interaction. (A) Localization pathogenic mutations p.Leu48Pro and p.Met104Val in the SANS molecule into human SANS. (B) Western blot analysis of GFP-Trap of FLAG-SANS, wild type, p.Leu48Pro and p.Met104Val, respectively, by GFP-tagged IFT52 and IFT57, immobilized on GFP-Trap beads. (C) Quantification of the recovery of FLAG-tagged SANS polypeptides by IFT-GFP-Traps indicating that the recovery of mutated SANS protein is drastically reduced. [file Image_1.JPEG]
